# Supplementary material for: The need for patient-centric medicine design: investigating key physical characteristics of oral solid medications to improve acceptance of older patients in Addis Ababa, Ethiopia
Source: PLoS One. 2026 Mar 6;21(3):e0331267. doi: 10.1371/journal.pone.0331267 (PMC13046391; doi:10.1371/journal.pone.0331267)
Supplement: S1 File — (DOCX) [file pone.0331267.s001.docx]

***Supplimantary material***

# Annex

**Consent Form**

I am informed fully in the language I understand about the aim of the above-mentioned research and other important points mentioned in the information sheet. I understood the purpose of the study entitled “The need of patient-centric medicine design: investigating key characteristics of oral solid medications to improve acceptance and adherence of elderly people in Addis Ababa, Ethiopia.”. I have been informed this study involves to explore the key characteristics of oral solid medications that can enhance acceptance and adherence among elderly patients. I have also read the information sheet. In addition, I have been told all the information collected through the research process is kept confidential. I understand my current and future medical services or other related issues will not be affected, if I refuse to participate or withdraw from the study.

I_______________________________, after being fully informed about the detail of this study, hereby gave my consent to participate in this study and approve my agreement with signature.

Participant name: _________________________ signature: _____Date: ________

Investigator name: _______________________ signature: _______ Date: _______

1. **Patient socio-demographic information**
2. Age: _______Year
3. Gender: A. Male B. Female
4. Religious Affiliation: A. Orthodox B. Muslim C. Protestant D. Other (please specify) ______________
5. Residence A. Rural B. Urban
6. Marital status A. Single B. Married C. Divorced D. Widowed E. Separated
7. Ethnicity A. Amhara B. Oromo C. Sidama D. Somali E. Other _____
8. Level of education A. No formal education B. Primary school C. Secondary school D. College or above
9. Occupation A. retired B. private work C. farmer D. Unemployed E. Other_______
10. Average monthly income? ________birr
11. Do you have health insurance A. Yes B. No
12. Do you have support from family members/care givers A. yes B. No
13. **Participants** **and Therapy Related Characteristics**
14. Alcohol consumption A. Non-drinker B. Occasional drinker C. Daily drinker
15. Tobacco smoking A. Nonsmoker B. Ex-smoker C. Smoker
16. Physical activity A. Yes B. No
17. Chat chewing A. chewer B. ex-chewer C. non- chewer
18. Do you think that you have good communication with health care provider? A. yes B. No
19. How do you describe your Functional dependency of daily living activities? A. Independent B. Slightly dependent C. Moderately dependent D. Severely dependent E. Totally dependent
20. Medication Dosing Reminders? A. Not using any reminder B. Use of alarms/phones/pillboxes C. Association of medication with daily routines D. family/caregivers
21. How long you started to take oral solid medications chronically? ________ years
22. Number of oral solid medications you currently taking? ______
23. **Ageing and medical disorder related factors**
24. What type of disorder have you experienced as you have gotten older?
25. Memory disorder
26. Swallowing disorder
27. Loose of sensation/touch
28. Visual impairment
29. Other (please specify) __________
30. Which medical disorder(s) are you currently being treated for with oral solid medication?
31. Hypertension B. Diabetes Mellitus (DM) C. Asthma D. Stroke E. Hyperlipidemia F. CKD G. Cancer H. Other (please specify) ________
32. How would you describe the severity of your current medical condition(s)?
33. Mild B. Moderate C. Severe
34. How many years has it been since you were first diagnosed with your current main medical condition? ________ years
35. How frequently do you visit medical centers to seek medical services in a year?
36. Monthly B. Every two-month C. Quarterly D. Biannually
37. How satisfied are you with your healthcare providers?
38. Very unsatisfied B. Unsatisfied C. Neutral C. Satisfied D. Very satisfied
39. How satisfied are you with quality of care you receive?
40. Very unsatisfied B. Unsatisfied C. Neutral C. Satisfied D. Very satisfied
41. **Behavioral related factors**
    1. **Elderly people belief about their oral solid medicine**

| NO | **General over use questions** | **SDA** | **DA** | **Neutral** | **A** | **SA** |
| --- | --- | --- | --- | --- | --- | --- |
| 1 | Doctors use too many oral solid medicines |  |  |  |  |  |
| 2 | Natural remedies are safer than oral solid medicines |  |  |  |  |  |
| 3 | Doctors place too much trust on oral solid medicines |  |  |  |  |  |
| 4 | If doctors had more time with patients, they would prescribe fewer oral solid medicines |  |  |  |  |  |
|  | **General harm belief questions** | **SDA** | **DA** | **Neutral** | **A** | **SA** |
| 1 | People who take oral solid medicines should stop their treatment for a while every now and again |  |  |  |  |  |
| 2 | Most oral solid medicines are addictive |  |  |  |  |  |
| 3 | Oral solid medicines do more harm than good |  |  |  |  |  |
| 4 | All oral solid medicines are poisons |  |  |  |  |  |
| NO | **Specific-Necessity Scale (Necessity beliefs) questions** | **SDA** | **DA** | **Neutral** | **A** | **SA** |
| 1 | My health, at present depends on my oral solid medicines |  |  |  |  |  |
| 2 | My life would be impossible without my oral solid medicines |  |  |  |  |  |
| 3 | Without my oral solid medicine, I would be very ill |  |  |  |  |  |
| 4 | My health in the future will depend on my oral solid medicines |  |  |  |  |  |
| 5 | My oral solid medicines protect me from becoming worse |  |  |  |  |  |
| NO | **Specific-Concerns Scale (Concern beliefs) questions** | **SDA** | **DA** | **Neutral** | **A** | **SA** |
| 1 | Having to take these oral solid medicines worries me |  |  |  |  |  |
| 2 | I sometimes worry about long-term effects of my oral solid medicines |  |  |  |  |  |
| 3 | My oral solid medicines are a mystery to me |  |  |  |  |  |
| 4 | My oral solid medicines disrupt my life |  |  |  |  |  |
| 5 | I sometimes worry about becoming too dependent on my oral solid medicines |  |  |  |  |  |

- 1. **Patient knowledge about taking oral solid medications**

| No | Knowledge about medicines | Choices | |
| --- | --- | --- | --- |
| 1 | What is your oral solid medicine name? | A | Know all his/her oral solid medication name |
|  |  | B | Know his/her oral solid medicines name partially |
|  |  | C | Does not know his/her oral solid medicines name |
| 2 | What is your oral solid medicine strength? | A | Know all his/her oral solid medication strength |
|  |  | B | Know his/her oral solid medicines strength partially |
|  |  | C | Does not know his/her oral solid medicines strength |
| 3 | For what purpose do you take these oral solid medicines? | A | Know the correct therapeutic indication |
|  |  | B | The information on therapeutic indication is not complete |
|  |  | C | Does not know the correct therapeutic indication partially |
|  |  | D | Does not know the correct therapeutic indications |
| 4 | At what time of day do you take your oral solid medicine? | A | Know the correct time for the administration of all his/her oral solid medicines |
|  |  | B | Know the correct time for the administration of his/her oral solid medicines partially |
|  |  | C | Does not know the correct time for administration of the oral solid medicine |
| 5 | How often do you take your oral solid medicines? | A | Know the correct time for dosage interval of all his/her oral solid medicine |
|  |  | B | Know the correct time for dosage interval of his /her oral solid medicines partially |
|  |  | C | Does not know the correct time for dosage interval of all his/her oral solid medicine |
| 6 | Where do you keep your oral solid medicines at home? | A | Know the correct storage conditions of each of his/her oral solid medicines |
|  |  | B | Lack of information on oral solid medication storage |
|  |  | C | Know the correct storage conditions of his /her oral solid medicines partially |
|  |  | D | Does not know the correct storage conditions of all of his/her oral solid medicines |

- 1. **Elderly peoples cultural and religious belief**

**Elderly people cultural beliefs related questions**

|  | Cultural belief statements about oral solid medications | SDA | DA | Neutral | A | SA |
| --- | --- | --- | --- | --- | --- | --- |
| 1 | In order for my disease condition to improve, I have to accumulate good deeds in my daily life. |  |  |  |  |  |
| 2 | My disease condition is a punishment for my bad behaviors |  |  |  |  |  |
| 3 | My disease condition is caused by witchcraft or evil eye |  |  |  |  |  |
| 4 | If people know I have my disease condition it will reflect badly not only myself but to my entire family |  |  |  |  |  |
| 5 | Other people play a big role in weather my disease condition improve, stay the same or gets worse |  |  |  |  |  |
| 6 | Luck plays a big part in determining how my disease condition improves |  |  |  |  |  |
| 7 | Following doctors order is the best way to keep my disease condition from getting any worse |  |  |  |  |  |
| 8 | My illness will improve by casting spell on food, drink or other objects |  |  |  |  |  |
| 9 | Traditional medicines are more effective for long standing disease than modern medicine. |  |  |  |  |  |

**Elderly people religious beliefs related questions**

|  | Spiritual perspective scale on oral solid medications | SDA | DA | N | A | SA |
| --- | --- | --- | --- | --- | --- | --- |
| 1 | In talking with family and friends, I often do my spiritual matters |  |  |  |  |  |
| 2 | I often share with others the problem and joys of living according to your spiritual beliefs |  |  |  |  |  |
| 3 | I often read spiritually related materials |  |  |  |  |  |
| 4 | I often engage in private prayer and medication |  |  |  |  |  |
| 5 | Forgiveness is an important part of my spirituality |  |  |  |  |  |
| 6 | I seek spiritual guidance in making decision in my everyday life |  |  |  |  |  |
| 7 | My spirituality is a significant part of my life |  |  |  |  |  |
| 8 | I frequently feel very close to god or higher power in prayer, during public worship or at important moment in my life |  |  |  |  |  |
| 9 | My spiritual views have had an influence upon my life |  |  |  |  |  |
| 10 | My spirituality is especially important because it answers many questions about the meaning of life |  |  |  |  |  |

1. **Oral solid medication related characteristics**

Please fill the following table with appropriate medication characteristics information.

| _S/N_ | _Name_ | _Size(mm)_ | _Shape_ | _Color_ | _Taste_ | _Scoring_ | _Packaging_ | _Texture_ | _Smell_ | _Form_ | _labeling_ |
| --- | --- | --- | --- | --- | --- | --- | --- | --- | --- | --- | --- |
| _1_ |  |  |  |  |  |  |  |  |  |  |  |
| _2_ |  |  |  |  |  |  |  |  |  |  |  |
| *_3_* |  |  |  |  |  |  |  |  |  |  |  |
| *_4_* |  |  |  |  |  |  |  |  |  |  |  |
| *_5_* |  |  |  |  |  |  |  |  |  |  |  |
| *_6_* |  |  |  |  |  |  |  |  |  |  |  |

1. ***Acceptance***

*For each of the following statements, please write the number of each of your medicine in the above table in a box that best describes your opinion (sd= strongly disagree, d=disagree, n=neutral, a=agree, sa= strongly agree)*

| ***This medicine -*** | ***Medication 1*** | | | | | ***Medication 2*** | | | | | ***Medication 3*** | | | | | ***Medication 4*** | | | | | | ***Medication 5*** | | | | |  |  |
| --- | --- | --- | --- | --- | --- | --- | --- | --- | --- | --- | --- | --- | --- | --- | --- | --- | --- | --- | --- | --- | --- | --- | --- | --- | --- | --- | --- | --- |
|  | *sd* | ***D*** | ***n*** | ***a*** | ***sa*** | ***sd*** | ***d*** | ***n*** | ***a*** | ***sa*** | ***Sd*** | ***D*** | ***n*** | ***a*** | ***sa*** | ***sd*** | ***D*** | | ***n*** | ***a*** | ***sa*** | ***sd*** | ***d*** | ***n*** | ***a*** | ***sa*** | | |
| *…has a convenient dose frequency. (E.g. once a day, twice a day or once weekly.)* |  |  |  |  |  |  |  |  |  |  |  |  |  |  |  |  |  | |  |  |  |  |  |  |  |  | |  |
| *…is a convenient amount for me to take. (E.g. number of tablets)* |  |  |  |  |  |  |  |  |  |  |  |  |  |  |  |  | |  |  |  |  |  |  |  |  |  |  |  |
| *…is easy to fit into my lifestyle.* |  |  |  |  |  |  |  |  |  |  |  |  |  |  |  |  | |  |  |  |  |  |  |  |  |  |  |  |
| *…is easy to take. (E.g. consider need for taking on an empty stomach or standing upright.)* |  |  |  |  |  |  |  |  |  |  |  |  |  |  |  |  | |  |  |  |  |  |  |  |  |  |  |  |
| *…is suitable to take when not at home. (E.g. consider need for water, or ease of carrying.)* |  |  |  |  |  |  |  |  |  |  |  |  |  |  |  |  | |  |  |  |  |  |  |  |  |  |  |  |
| *…tastes good.* |  |  |  |  |  |  |  |  |  |  |  |  |  |  |  |  | |  |  |  |  |  |  |  |  |  |  |  |
| *...has no bad aftertaste* |  |  |  |  |  |  |  |  |  |  |  |  |  |  |  |  | |  |  |  |  |  |  |  |  |  |  |  |
| *…has a good texture. (E.g. Smooth or fizzy.)* |  |  |  |  |  |  |  |  |  |  |  |  |  |  |  |  | |  |  |  |  |  |  |  |  |  |  |  |
| *… is easy to swallow (right size).* |  |  |  |  |  |  |  |  |  |  |  |  |  |  |  |  | |  |  |  |  |  |  |  |  |  |  |  |
| *… is easy to swallow (right shape).* |  |  |  |  |  |  |  |  |  |  |  |  |  |  |  |  | |  |  |  |  |  |  |  |  |  |  |  |
| *… is easy to swallow (right thickness).* |  |  |  |  |  |  |  |  |  |  |  |  |  |  |  |  | |  |  |  |  |  |  |  |  |  |  |  |
| *… is easy to use without modifications* |  |  |  |  |  |  |  |  |  |  |  |  |  |  |  |  | |  |  |  |  |  |  |  |  |  |  |  |
| *…is easy to see.* |  |  |  |  |  |  |  |  |  |  |  |  |  |  |  |  | |  |  |  |  |  |  |  |  |  |  |  |
| *…has a nice color* |  |  |  |  |  |  |  |  |  |  |  |  |  |  |  |  | |  |  |  |  |  |  |  |  |  |  |  |
| *…has no bad smell* |  |  |  |  |  |  |  |  |  |  |  |  |  |  |  |  | |  |  |  |  |  |  |  |  |  |  |  |
| *…is easy to take out of its packaging.* |  |  |  |  |  |  |  |  |  |  |  |  |  |  |  |  | |  |  |  |  |  |  |  |  |  |  |  |
| *…is easy to hold.* |  |  |  |  |  |  |  |  |  |  |  |  |  |  |  |  | |  |  |  |  |  |  |  |  |  |  |  |
| *…does what it is supposed to do. (E.g. helps my symptoms or prevents further illness.)* |  |  |  |  |  |  |  |  |  |  |  |  |  |  |  |  | |  |  |  |  |  |  |  |  |  |  |  |
| *…works quickly.* |  |  |  |  |  |  |  |  |  |  |  |  |  |  |  |  | |  |  |  |  |  |  |  |  |  |  |  |
| *…makes me feel better.* |  |  |  |  |  |  |  |  |  |  |  |  |  |  |  |  | |  |  |  |  |  |  |  |  |  |  |  |
| *…makes me feel ill all the time* |  |  |  |  |  |  |  |  |  |  |  |  |  |  |  |  | |  |  |  |  |  |  |  |  |  |  |  |
| *…makes me feel ill for a short time just after taking it.* |  |  |  |  |  |  |  |  |  |  |  |  |  |  |  |  | |  |  |  |  |  |  |  |  |  |  |  |

**አባሪ**

**የስምምነት ቅጽ**

ከላይ ስለተጠቀሰው ምርምር ዓላማ እና በመረጃ ወረቀቱ ላይ ስለተጠቀሱ ሌሎች ጠቃሚ ነጥቦች በምረዳው ቋንቋ ሙሉ መረጃ አግኝቻለሁ። የጥናቱ ዓላማ “ታካሚዎችን ያማከለ የመድኃኒት ዲዛይን አስፈላጊነት፡ በአዲስ አበባ፣ ኢትዮጵያ ውስጥ ያሉ አረጋውያንን መዲሃኒት መቀበልን እና ተደራሽነትን ለማሻሻል በአፍ የሚወሰዱ ጠጣር የመድኃኒቶችን ቁልፍ ባህሪያት መመርመር” እንደሆነ ተረድቻለሁ። ይህ ጥናት በአረጋውያን መካከል መዲሃኒት መቀበልን እና ተደራሽነትን ሊያሳድጉ የሚችሉ በአፍ የሚወሰዱ ጠጣር የመድኃኒቶችን ዋና ዋና ባህሪያትን መመርመርን እንደሚያካትት ተረድቻለሁ። የመረጃ ወረቀቱንም አንብቤዋለሁ። በተጨማሪም፣ በምርምር ሂደቱ የሚሰበሰቡት መረጃዎች በሙሉ በሚስጥር እንደሚጠበቁ ተነግሮኛል። ከጥናቱ ለመሳተፍ ወይም ለመካፈል ፈቃደኛ ካልሆንኩ የአሁኑ እና የወደፊት የሕክምና አገልግሎቶቼ ወይም ሌሎች ተዛማጅ ጉዳዮች እንደማይጎዱ ተረድቻለሁ።

እኔ _______________________________፣ ስለዚህ ጥናት ዝርዝር ሙሉ በሙሉ ከተረዳሁ በኋላ፣ በዚህ ጥናት ላይ ለመሳተፍ ፈቃዴን ሰጥቼ ፊርማዬን አጽድቄያለሁ።

የተሳታፊ ስም፡ ___________________ ፊርማ፡ _____ቀን፡ ________

የተመራማሪው ስም፡- _______________________ ፊርማ፡_______ ቀን፡ _______

1. **የተሳታፊዎች ሶሽዮዲሞግራፊክ መረጃዎች**
2. እድሜ፤ _____________
3. ጾታ፤ ሀ) ወንድ ለ) ሴት
4. ሀይማኖት፤ ሀ) ኦርቶዶክስ ለ) ሙስሊም መ) ፕሮቴስታንት ሰ) ሌላ (ጥቀስ)______________
5. የመኖሪያ ቦታ፤ ሀ) ገጠር ለ) ከተማ
6. የጋብቻ ሁኔታ፤ ሀ) ያላገባ(ች) ለ)ያገባ(ች) መ)የፈታ(ች) ሰ) በሞት የተለየ(ች)
7. የአፍ መፍቻ ቋንቋ፤ ሀ) አማረኛ ለ)ኦሮመኛ መ)ትግረኛ ሰ)ሶማለኛ ረ) ሌላ ________________
8. የትምህርት ደረጃ፤ ሀ) መደበኛ ትምህርት የለም ለ)አንደኛ ደረጃ መ) ሁለተኛ ደረጃ ሰ)ኮሌጅ እና ከዛ በላይ
9. የስራ ሁኔታ ሀ) ጡረተኛ ለ) የግል ስራ መ)ገበሬ ሰ) ስራ የሌለዉ ረ) ሌላ ________________
10. አማካኝ ወርሀዊ ገቢ ____________
11. የጤና መድህን አለዎት? ሀ) አዎ ለ) የለኝም
12. ከበተሰበዎ ወይም ከድጋፍ ሰጭ ሰራተኛዎ ድጋፍ ያገኛሉ? ሀ) አዎ ለ) የለኝም
13. **ከተሳታፊዎች እና ህክምና ጋር የተገናኙ ባህሪያት**
14. አልኮል ይጠጣሉ? ሀ) አልጠጣም ለ) አንዳንደ እጠጣለሁ መ) በየቀኑ እጠጣለሁ
15. ሲጋራ ያጨሳሉ? ሀ) አላጨስም ለ) በፊት አጨስ ነበር መ) አጨሳለሁ
16. ጫት ይቅማሉ? ሀ) አልቅምም ለ) በፊት እቅም ነበር መ)እቅማለሁ
17. ከጤና ባለሙያዎች ጋር ጥሩ ተግባቦት አለኝ ብለው ያስባሉ ሀ) አዎ ለ) የለኝም
18. እለት ከእለት ተግባረዎ ላይ ያለዎትን የሰዎችን እርዳታ መፈለግ እንደት ይገልጹታል? ሀ) ራሴን እችላለሁ ለ) በትንሹ እፈልጋለሁ መ) በመካከለኛ ደረጃ እፈልጋለሁ ሰ) በጣም እፈልጋለሁ ረ) ሙሉ በሙሉ እፈልጋለሁ
19. የመድሃኒት መዉሰጃ ሰአተዎ ሲደርስ የሚያስታዉሰዎት ነገር ዪጠቅማሉ? ሀ) ምንም ነገር አልጥቀምም ለ) አላርም/ስልክ እጠቀማለሁ መ) መድሀኒቴን ከእለታዊ ከማደርጋቸዉ ነገሮች ጋር በማቅናጀት ሰ)ቤተሰብ/ሰራተኛ
20. በአፍ የሚዎሰዱ ኪነኖችን መዉሰድ ከጀመሩ ምን ያክል ጊዜ ይሆነወታል? _________አመት
21. በአሁኑ ሰአት ምን ያክል በአፍ የሚዎሰዱ ኪነኖችን ይዎስዳሉ? _________________
22. **ከእድሜ እና ከህክምና ጋር የተገናኙ የጤና ችግሮች**
23. እድሜዎ እየጨመረ ሲመጣ ከእድሜ ጋር የተገናኙ ምን አይነት ችግሮች አጋጥሞዎታል?

ሀ) የማስታዎስ ችግር ለ) የመዋጥ ችግር መ) በመንካት ጊዜ ስሜት አለመሰማት ሰ) የእይታ መጥን መቅንስ ረ) ሌላ (ጥቅስ) _____________

1. በአሁኑ ሰአት መድሀኒት እየዎሰዱ ያሉት ለየትኛው አይንት ህመምዎ ነው? ሀ) የደም ግፊት ለ) ስኳር

መ) አስም ሰ) ስትሮክ ረ) ስብ ሸ) ኩላሊት ቀ) ካንሰር በ) ሌላ(ጥቅስ) ______________________

1. አሁን ያለዉን የህመመዎን ክብደት እንደት ይገልጹታል? ሀ) ቀላል ለ) መካከለኛ መ) ከባድ
2. አሁን ካሉበዎት የህመም አይነቶች መካከል የመጀመሪያዉ ህመም እንዳለበዎት ካዎቁ ምን ያክል ይሆነዎታል?______________አመት
3. በአመት ዉስጥ የህክምና አገልግሎት ለማገኘት ምን ያክል ጊዜ ወደ ጤና ተቋማት ይሄዳሉ?
4. በጤና ባለሙያዎች አገልግሎት ምን ያክል እረክተዋል?

ሀ) በጣም አረካሁም ለ) አረካሁም መ) ገለልተኛ ሰ) እረክቻለሁ ረ) በጣም እረክቻለሁ

1. ከጤና ተቋማቱ ባገኙት የእንክብካቤ ጥራት ምን ያክል ረክተዋል?

ሀ) በጣም አረካሁም ለ) አረካሁም መ) ገለልተኛ ሰ) እረክቻለሁ ረ) በጣም እረክቻለሁ

1. ከግለሰቡ ባህሪ ጋር የተገናኙ ጥያቄዎች

| ተ/ቁ | **ከተገቢዉ በላይ መጠቀምን የተመለከቱ ጥያቄዎች** | **በ/አል** | **አል** | **ገለልተኛ** | **እስ** | **በ/እስ** |
| --- | --- | --- | --- | --- | --- | --- |
| 1 | ሃኪሞች በጣም ብዙ በአፍ የሚዎሰዱ ጠጣር መድሃኒቶችን ያዛሉ። |  |  |  |  |  |
| 2 | በአፍ ከሚዎሰዱ ጠጣር ዘመናዊ መዲሃኒቶች ይልቅ ተፈጥሮአዊ መድሃኒቶች ደህንነታቸዉ አስተማማኝ ነው። |  |  |  |  |  |
| 3 | ሃኪሞች በአፍ የሚዎሰዱ ጠጣር መድሃኒቶች ላይ በጣም ከፍተኛ እምነት አላቸው። |  |  |  |  |  |
| 4 | ሃኪሞች ከታካሚዎች ጋር ብዙ ጊዜ ካሳለፉ፣ ጥቂት በአፍ የሚዎሰዱ ጠጣር መድሃኒቶችን ብቻ ሊያዙላቸዉ ይችላሉ። |  |  |  |  |  |
|  | **አጠቃላይ ስለመዳኒቶች ጉዳት ያለዎት እምነት** | **በ/አል** | **አል** | **ገለልተኛ** | **እስ** | **በ/እስ** |
| 1 | በአፍ የሚዎሰዱ ጠጣር መድሃኒቶችን የሚዎስዱ ሰዎች አልፎ አልፎ ለተወሰነ ጊዜ መድሃኒት መዉሰዳቸዉን ማቆም አለባቸዉ። |  |  |  |  |  |
| 2 | አብዛሃኞቹ በአፍ የሚዎሰዱ ጠጣር መድሃኒቶች ጥገኝነትን ይፈጥራሉ። |  |  |  |  |  |
| 3 | በአፍ የሚዎሰዱ ጠጣር መድሃኒቶች ከጥቅማቸዉ ጉዳታቸዉ ያመዝናል። |  |  |  |  |  |
| 4 | ሁሉም በአፍ የሚዎሰዱ ጠጣር መድሃኒቶች መርዛማ ናቸዉ |  |  |  |  |  |
| NO | **በመድሃኒቶቹ ላይ ያለዎት ልዩ የአስፈላጊነት እምነት** | **በ/አል** | **አል** | **ገለልተኛ** | **እስ** | **በ/እስ** |
| 1 | ጤናየ በአሁኑ ሰአት በአፍ የሚዎሰዱ ጠጣር መድሃኒቶች ላይ ጥገኛ ነው። |  |  |  |  |  |
| 2 | በአፍ የሚዎሰዱ ጠጣር መድሃኒቶች ባይኖሩ ኖሮ ህይዎቴን ማቆየት አይቻልም ነበር። |  |  |  |  |  |
| 3 | በአፍ የሚዎሰዱ ጠጣር መድሃኒቶች ባይኖሩ ኖሮ በጣም እታመም ነበር። |  |  |  |  |  |
| 4 | ጤናየ ወደፊት በአፍ የሚዎሰዱ ጠጣር መድሃኒቶች ላይ ጥገኛ ይሆናል። |  |  |  |  |  |
| 5 | የእኔ በአፍ የሚዎሰዱ ጠጣር መድሃኒቶች ህመሜ እንዳይባባስ ተከላክለዉልኛል። |  |  |  |  |  |
| NO | **በመድሃኒቶቹ ላይ ያለዎት ልዩ የቅሬታ እምነቶች** | **በ/አል** | **አል** | **ገለልተኛ** | **እስ** | **በ/እስ** |
| 1 | እነዚህን በአፍ የሚዎሰዱ ጠጣር መድሃኒቶች መዉሰደ እያስጨነቀኝ ነዉ። |  |  |  |  |  |
| 2 | አንድ አንድ ጊዜ በአፍ የሚዎሰዱ ጠጣር መድሃኒቶች ስለእረጂም ጊዜ ጉዳታቸዉ እጨነቃለሁ። |  |  |  |  |  |
| 3 | በአፍ የምወስዳቸዉ ጠጣር መድሃኒቶች ለእኔ ሚስጥር ናቸዉ። |  |  |  |  |  |
| 4 | በአፍ የምወስዳችዉ ጠጣር መድሃኒቶች ህይወቴን እረብሸዉብኛል። |  |  |  |  |  |
| 5 | አንድ አንድ ጊዜ በአፍ የሚዎሰዱ ጠጣር መድሃኒቶች ላይ በጣም ጥገኛ እየሆንኩ መምጣቴ ያስጭንቀኛል። |  |  |  |  |  |

1. **ታካሚውች በአፍ ስለሚዎስዷቸው መድሃኒቶች ያላቸዉ እዉቀት**

| ተ/ቁ | እዉቀት(ስለመድሃኒተዎ) | ምርቻዎች | |
| --- | --- | --- | --- |
| 1 | በአፈዎ የሚወስዷቸዉን ጠጣር መዲሃኒቶች ስም ይዘርዝሩ | A | ሁሉንም በአፍ የሚዎስዱትን ጠጣር መድሃኒት ስሞች ያዉቃሉ |
|  |  | B | በአፍ የሚዎስዱትን ጠጣርመድሃኒት ስሞች በከፊል ያዉቃሉ |
|  |  | C | ሁሉንም በአፍ የሚዎስዱትን ጠጣር መድሃኒት ስሞች አያዉቁም |
| 2 | በአፈዎ የሚወስዷቸዉ መዲሃኒቶች ጥንካሬያቸዉ ምን ያክል እንደሆነ ያዉቃሉ? | A | ሁሉንም በአፍ የሚዎስዱትን ጠጣር መድሃኒቶች ጥንካሬያቸዉን ያዉቃሉ |
|  |  | B | በአፍ የሚዎስዱትን ጠጣር መድሃኒቶች ጥንካሬያቸዉን በከፊል ያዉቃሉ |
|  |  | C | ሁሉንም በአፍ የሚዎስዱትን ጠጣር መድሃኒቶች ጥንካሬያቸዉን አያዉቁም |
| 3 | እነዚህን በአፍ የሚዎሰዱ መድሃኒቶች ለምን አላማ እንደሚዎስዷቸዉ ያዉቃሉ? | A | ሁሉንም መድሃኒቶች ለምን አላማ እንደሚውወስዷቸዉ ያዉቃሉ |
|  |  | B | መዲሃኒቶቹ ለምን አላማ እንደምወስዳቸዉ ግልጥ የሆነ የተሟላ መረጃ የለኝም |
|  |  | C | መድሃኒቶችን ለምን አላማ እንደሚውወስዷቸዉ በከፊል ያዉቃሉ |
|  |  | D | ሁሉንም መድሃኒቶች ለምን አላማ እንደሚውወስዷቸዉ አያዉቁም |
| 4 | እነዚህን በአፍ የሚዎሰዱ መድሃኒቶች በቀን ዉስጥ በየትኛው ሰአት ይውስዳሉ? | A | ሁሉንም በአፍ የሚወሰዱ ጠጣር መድሃኒቶች በየትኛው ሰአት እንደሚወስዷቸው ያዉቃሉ |
|  |  | B | በአፍ የሚወሰዱ ጠጣር መድሃኒቶች በየትኛው ሰአት እንደሚወስዷቸው በከፊል ያዉቃሉ |
|  |  | C | ሁሉንም በአፍ የሚወሰዱ ጠጣር መድሃኒቶች በየትኛው ሰአት እንደሚወስዷቸው አያዉቁም |
| 5 | እነዚህን በአፍ የሚዎሰዱ መድሃኒቶችን በቀን ምን ያክል ጊዜ እንደሚወሰዱ ያዉቃሉ? | A | ሁሉንም በአፍ የሚዎሰዱ ጠጣር መድሃኒቶች ምን ያክል ጊዜ እንደሚወሰዱ ያዉቃሉ |
|  |  | B | በአፍ የሚዎሰዱ ጠጣር መድሃኒቶች ምን ያክል ጊዜ እንደሚወሰዱ በከፊል ያዉቃሉ |
|  |  | C | ሁሉንም በአፍ የሚዎሰዱ ጠጣር መድሃኒቶች ምን ያክል ጊዜ እንደሚወሰዱ አያዉቁም |
| 6 | እነዚህን በአፍ የሚዎሰዱ መድሃኒቶችን ቤትዎ ዉስጥ የት ያስቀምጣሉ? | A | ሁሉንም በአፍ የሚዎሰዱ ጠጣር መድሃኒቶች ቤት ዉስጥ የት እንደሚቀመጡ ያዉቃሉ |
|  |  | B | መዲሃኒቶቹ ቤት ዉስጥ የት እንደሚቅመጡ መረጃ የላቸዉም |
|  |  | C | ሁሉንም በአፍ የሚዎሰዱ ጠጣር መድሃኒቶች ቤት ዉስጥ የት እንደሚቀመጡ በከፊል ያዉቃሉ |
|  |  | D | ሁሉንም በአፍ የሚዎሰዱ ጠጣር መድሃኒቶች ቤት ዉስጥ የት እንደሚቀመጡ አያዉቁም |

1. **የታካሚዎች የባህላዊ እና ሃይማኖታዊ እምነቶችን የተመለከቱ ጥያቄውች**

**የታካሚዎች የባህላዊ እምነቶችን የተመለከቱ ጥያቄው**

|  | የታካሚዎች የባህላዊ እምነቶችን የተመለከቱ አረፍተነገሮች | **በ/አል** | **አል** | **ገለልተኛ** | **እስ** | **በ/እስ** |
| --- | --- | --- | --- | --- | --- | --- |
| 1 | የበሽታዬ ሁኔታ እንዲሻሻል, በዕለት ከዕለት ሕይወቴ ውስጥ መልካም ስራዎችን ማከማቸት አለብኝ |  |  |  |  |  |
| 2 | የበሽታዬ ሁኔታ ለመጥፎ ባህሪዎቼ ቅጣት ነው። |  |  |  |  |  |
| 3 | የበሽታዬ ሁኔታ በጥንቆላ ወይም በዓይነ፟ ጥላ ምክንያት የመጣ ነው። |  |  |  |  |  |
| 4 | ሰዎች ያለብኝን የበሽታዬን ሁኔታ ካወቁ እኔን ብቻ ሳይሆን መላው ቤተሰቤ ላይ መጥፎ ነገርን ያንፀባርቃል |  |  |  |  |  |
| 5 | ሌሎች ሰዎች የእኔ በሽታ ሁኔታ እንዲሻሻል, ተመሳሳይ ሆኖ እንዲቆይ ወይም እንዲባባስ ትልቅ ሚና ይጫወታሉ |  |  |  |  |  |
| 6 | የበሽታዬ ሁኔታ እንዴት እንደሚሻሻል ለመወሰን ዕድል ትልቅ ሚና ይጫወታል |  |  |  |  |  |
| 7 | የበሽታዬ ሁኔታ እንዳይባባስ ለመከላከል የዶክተሮች ትእዛዝን መከተል ምርጡ መንገድ ነው። |  |  |  |  |  |
| 8 | ህመሜ በምግብ፣ መጠጥ ወይም ሌሎች ነገሮች ላይ ድግምት ወይም ጸሎት በማድረግ ይሻሻላል። |  |  |  |  |  |
| 9 | ባህላዊ መድሃኒቶች ከዘመናዊው መድሃኒት ይልቅ ለረጅም ጊዜ ለሚቆዩ በሽታዎች የበለጠ ውጤታማ ናቸው |  |  |  |  |  |

**የታካሚዎች ሃይማኖታዊ እምነቶች ጋር ተዛማጅ የሆኑ ጥያቄዎች**

|  | በአፍ የሚወሰድ ጠጣር መድሃኒቶች ላይ የመንፈሳዊ/ሃይማኖታዊ እይታ ልኬት | SDA | DA | N | A | SA |
| --- | --- | --- | --- | --- | --- | --- |
| 1 | ከቤተሰቦቼና ከጓደኞቼ ጋር ሳወራ ብዙ ጊዜ መንፈሳዊ ጉዳዮቼን እፈጽማለሁ/አነሳለሁ |  |  |  |  |  |
| 2 | በመንፈሳዊ ህይወት ዉስጥ ስኖር የሚያጋጥመኝን ችግር እና ደስታ ለሌሎች ብዙ ጊዜ አካፈላለሁ። |  |  |  |  |  |
| 3 | ከመንፈሳዊ ጉዳዮች ጋር የተያያዙ ጽሑፎችን ብዙ ጊዜ አነባለሁ። |  |  |  |  |  |
| 4 | ብዙ ጊዜ በግል ጸሎትና ህክምና ላይ አሳሊፋለሁ። |  |  |  |  |  |
| 5 | ይቅርታ አድራጊነት የመንፈሳዊነቴ አስፈላጊዉ አካል ነው። |  |  |  |  |  |
| 6 | በዕለት ከዕለት ሕይወቴ ውሳኔ ለማድረግ መንፈሳዊ መመሪያን እሻለሁ። |  |  |  |  |  |
| 7 | መንፈሳዊነቴ የሕይወቴ ጉልህ ክፍል ነው። |  |  |  |  |  |
| 8 | በጸሎት፣ በሕዝብ አምልኮ ወቅት ወይም በሕይወቴ አስፈላጊ በሆነ ወቅት ብዙ ጊዜ ወደ አምላኬ በጣም የተጠጋሁ ይሰማኛል። |  |  |  |  |  |
| 9 | መንፈሳዊ አመለካከቴ በሕይወቴ ላይ ተጽዕኖ አሳድሯል። |  |  |  |  |  |
| 10 | የእኔ መንፈሳዊነት በተለይ ስለ ሕይወት ትርጉም ብዙ ጥያቄዎችን ስለሚመልስ በጣም አስፈላጊ ነው። |  |  |  |  |  |

1. **በአፍ የሚወሰዱ ጠጣር መድሀኒቶችን ባህሪ የተመለከቱ ጥያቄዎች**

እባክዎ የሚከተለውን ሰንጠረዥ በተገቢው የመድሃኒት ባህሪያት መረጃ ይሙሉ

| ተ/ቁ | ስም | መጠን(ሚሚ) | ቅርጽ | ቀለም | ጣእም | መክፈያ መስመር | ማሸጊያ | ሲሪት | ምልክት | ሽታ | አይነት |
| --- | --- | --- | --- | --- | --- | --- | --- | --- | --- | --- | --- |
| 1 |  |  |  |  |  |  |  |  |  |  |  |
| 2 |  |  |  |  |  |  |  |  |  |  |  |
| 3 |  |  |  |  |  |  |  |  |  |  |  |
| 4 |  |  |  |  |  |  |  |  |  |  |  |
| 5 |  |  |  |  |  |  |  |  |  |  |  |
| 6 |  |  |  |  |  |  |  |  |  |  |  |

1. **በአፍ የሚዎሰዱ ጠጣር መድሃኒቶች ያላችዉ ተቀባይነት**

ለእያንዳንዱ የሚከተሉት መግለጫዎች፣ እባክዎ የእያንዳንዱን መድሃኒትዎን ቁጥር(ከዚህ በላይ ባለው ሠንጠረዥ አሞላል ቅደም ተከተል መሰረት) የእርስዎን ልምድ እና አስተያየት በተሻለ ሁኔታ በሚገልጽ ሳጥን ውስጥ ይፃፉ። እያንዳንዱ መድሀኒት በእያንዳንዱ ሳጥን ዉስጥ ይሞላል።

| **ይህ መድሀኒት** | **በጣም አልስማማም** | **አልስማማም** | **ገለልትኛ** | **እስማማለሁ** | **በጣም እስማማለሁ** |
| --- | --- | --- | --- | --- | --- |
| … ምቹ የመጠን ድግግሞሽ አለው። (ለምሳሌ በቀን አንድ ጊዜ፣ በቀን ሁለት ጊዜ ወይም በሳምንት አንድ ጊዜ።) |  |  |  |  |  |
| ... ለመውሰድ ምቹ መጠን ነው። (ለምሳሌ የኪኒኖች ብዛት) |  |  |  |  |  |
| …ከአኗኗሬ ጋር ለመስማማት ቀላል ነው። |  |  |  |  |  |
| ... ለመውሰድ ቀላል ነው። (ለምሳሌ ባዶ ሆድ መውሰድ ወይም ቀና ብሎ መቆምን ያስቡት።) |  |  |  |  |  |
| ... ቤት ውስጥ ባልሆን እንኳ ለመውሰድ ተስማሚ ነው. (ለምሳሌ የውሃ ፍላጎትን ወይም ይዞ ለመንቅሳቀስ ያለዉን ቀላልነት ግምት ውስጥ ያስገቡ።) |  |  |  |  |  |
| ...ጥሩ ጣእም አለዉ። |  |  |  |  |  |
| ... ከዋጥኩት በኋላ መጥፎ ጣዕም የለውም |  |  |  |  |  |
| …ጥሩ ስሪት አለው። (ለምሳሌ ለስላሳ) |  |  |  |  |  |
| … ለመዋጥ ቀላል ነው (ትክክለኛ መጠን አለው)። |  |  |  |  |  |
| … ለመዋጥ ቀላል ነው (ትክክለኛ ቅርጽ አለው)። |  |  |  |  |  |
| … ለመዋጥ ቀላል ነው (ትክክለኛ መጠን አለው)። |  |  |  |  |  |
| … ያለምንም ማስተካከያ ለመዉሰድ ቀላል ነዉ |  |  |  |  |  |
| …ለማየት ቀላል ነዉ |  |  |  |  |  |
| … ጥሩ ቀለም አለው። |  |  |  |  |  |
| … መጥፎ ጠረን የለውም |  |  |  |  |  |
| …ከማሸጊያው ለማውጣት ቀላል ነው። |  |  |  |  |  |
| ... ለመያዝ ቀላል ነው። |  |  |  |  |  |
| … ማድረግ ያለበትን ያደርጋል። (ለምሳሌ የበሽታዉን ምልክት ያጥፋል ወይም ተጨማሪ በሽታን ይከላከላል።) |  |  |  |  |  |
| … በፍጥነት ይሰራል። |  |  |  |  |  |
| ... ጥሩ ስሜት እንዲሰማኝ ያደርጋል። |  |  |  |  |  |
| …ሁልጊዜ ህመም እንዲሰማኝ ያደርጋል |  |  |  |  |  |
| …ከወሰድኩ በኋላ ለአጭር ጊዜ ህመም እንዲሰማኝ ያደርገኛል። |  |  |  |  |  |
