# Supplementary material for: The need for patient-centric medicine design: investigating key physical characteristics of oral solid medications to improve acceptance of older patients in Addis Ababa, Ethiopia
Source: PLoS One. 2026 Mar 6;21(3):e0331267. doi: 10.1371/journal.pone.0331267 (PMC13046391; doi:10.1371/journal.pone.0331267)
Supplement: S3 File — (PDF) [file pone.0331267.s003.pdf]

S3 Codebook

Variable Values		
Value		Label
agecatagory	1.00	60-64
	2.00	65-69
	3.00	70-74
	4.00	75-79
	5.00	80-84
	6.00	85-89
	7.00	greater than 80
@_2_Sex	1	Male
	2	female
@_3_Religious_Affiliation	1	orthodox
	2	muslim
	3	protestant
	4	other
@_4_Residence	1	urban
	2	Rural
@_5_Marital_status	1	married
	2	widowed
	3	single
	4	separated

	5	divorced
region	1	Amhara
	2	Addis Ababa
	3	Oromia
	4	Tigray
	5	Southern Ethiopian Religions
	6	others
@_7_Level_of_education	1	no formal education(illitrate
	2	primary school
	3	secondary school
	4	college and above
@_8_Occupation	1	retired
	2	private work
	3	unemployed
	4	farmer
	5	other
income	1.00	less than or equal to 2000
	2.00	2001-5000
	3.00	more than 5000
	1	Yes

@_10_Do_you_have_health_insurance	2	No
@_11_Do_you_have_suppo_members_care_givers	1	Yes
	2	No
@_1_Alcohol_consumption	1	non drinker
	2	ocasionally dirinker
	3	daily drinker
@_2_Tobacco_smoking	1	none smoker
	2	ex smoker
	3	smoker
@_3_Physical_activity	1	Yes
	2	No
@_4_Chat_chewing	1	non chewer
	2	ex-chewer
	3	chewer
@_5_Do_you_think_that_health_care_provider	1	Yes
	2	No
@_6_How_do_you_descri_ly_living_activities	1	independent
	2	slightly dependent
	3	moderetly dependant
	4	severly dependent

	5	totally dependant
@_7_Medication_Dosing_Reminders	1	no reminder
	2	family/caregiver
	3	allarm
	4	daily routin
yearsr	1.00	less than or equal to 5 years
	2.00	5-10
	3.00	10-15
	4.00	>15
Memory problem	.00	No
	1.00	Yes
Swallowing problem	.00	No
	1.00	Yes
Visual problem	.00	No
	1.00	Yes
Movement problem	.00	No
	1.00	Yes
Sensation problem	.00	No
	1.00	Yes
Hypertension	1.00	No
	2.00	Yes

DM	1.00	No
	2.00	Yes
Cardiac	1.00	No
	2.00	Yes
Asthma	1.00	No
	2.00	Yes
Inflammation	1.00	No
	2.00	Yes
CNS	1.00	No
	2.00	Yes
Endocrine	1.00	No
	2.00	Yes
CLD	1.00	No
	2.00	Yes
Infection	1.00	No
	2.00	Yes
Other	1.00	No
	2.00	Yes
@_3_How_would_you_des_medical_condition_s	1	Mild
	2	moderate
	3	severe
	1	monthly

@_5_How_frequently_do_l_services_in_a_year	2	every two month
	3	quarterly
	4	bi anually
@_6_How_satisfied_are_healthcare_providers	1	strongly unsatisfied
	2	unsatisfied
	3	neutral
	4	satisfied
	5	strongly satisfied
@_7_How_satisfied_are_of_care_you_receive	1	strongly unsatisfied
	2	unsatisfied
	3	neutral
	4	satisfied
	5	strongly satisfied
General over use belief	.00	weak believers
	1.00	strong believres
General harm belief	.00	Weak believer
	1.00	strong believres
Necessity belief	.00	Weak believer
	1.00	strong believres
Concern belief	.00	Weak believer
	1.00	strong believres
Religious belief	.00	Weak believer

	1.00	strong believres
caltural_belief	.00	Weak believer
	1.00	Fair believer
	2.00	strong believres
adhere0002	1.00	less than 28
	2.00	28
Knowledge	.00	Poor
	1.00	Good
Size	1.00	less than or equal to 6mm
	2.00	6.01-9.00mm
	3.00	9.01-12.00mm
	4.00	12.01-15.00mm
	5.00	15.01-18.00mm
	6.00	graeter than 18mm
Shape	1	round
	2	Oval
	3	oblong
Color	1	white
	2	yellow
	3	two or more color
	4	orange/green

	5	pink/peach/red
	6	blue
Taste	1	bitter
	2	sweet/fruity
	3	neutral
	4	salty/sour/metalic
Scoreline	1	none
	2	single
	3	partial
	4	multiple
Package	1.00	blister
	2.00	child reseistant bottle
	3.00	non child reseastant bottle
	4.00	plastic sack
Texture	1.00	coated/smooth
	2.00	uncoated/rough
	3.00	chewable/disintegrating
Smell	1.00	good
	2.00	bad
	3.00	neutral

Form	1.00	tablet
	2.00	capsule
Labling	1.00	not understand the language
	2.00	difficult to see and understand
	3.00	ease to see and understand
acceptance	1.00	No
	2.00	Yes
