# Supplementary material for: The need for patient-centric medicine design: investigating key physical characteristics of oral solid medications to improve acceptance of older patients in Addis Ababa, Ethiopia
Source: PLoS One. 2026 Mar 6;21(3):e0331267. doi: 10.1371/journal.pone.0331267 (PMC13046391; doi:10.1371/journal.pone.0331267)
Supplement: Attachment — Submitted filename: Response to Reviewers.docx [file pone.0331267.s005.docx]

**Response to Reviewers**

**Dear Reviewers and Editors,**

Thank you for your constructive feedback and valuable suggestions. We have addressed all the comments and questions raised in the revised manuscript and provided detailed responses in the separate rebuttal document.

| **S/N** | **Question/comment** | **Response** |
| --- | --- | --- |
| 1 | Is the manuscript technically sound, and do the data support the conclusions? | In my opinion *Yes.* The manuscript is technically sound. The data, including logistic regression analyses of medication acceptance by elderly patients, clearly support the conclusions regarding the influence of tablet/capsule size, coating, score lines, and labeling on patient acceptance, while also considering sociodemographic, clinical, and behavioral factors. |
| 2 | Has the statistical analysis been performed appropriately and rigorously? | Yes. The statistical analysis is appropriate and rigorous. Bivariable and multivariable logistic regression were used to identify factors associated with medication acceptance, with cluster-robust standard errors accounting for multiple medications per patient. Model assumptions, multicollinearity, calibration, discrimination, and influence were assessed, and missing data (<5%) were handled via complete-case analysis. |
| **3** | Have the authors made all data underlying the findings in their manuscript fully available? | Not, I will make it available now |
|  | Is the manuscript presented in an intelligible fashion and written in standard English? | **Yes** |
|  | Limit the use of abbreviations which are not frequently used or standardized. Explain Abbreviations and add statistical test in the footnote of tables. Remove “2025” from the title of tables..etc | Thank you for the comments; we have carefully considered them and revised the manuscript accordingly. All non-standard abbreviations have been minimized and defined at first use, statistical tests have been added to the footnotes of tables, and the year “2025” has been removed from table titles. |
|  | **Abstract** • Mention the knowledge gap in the. • Clarify in the abstract the type of data analysed i.e. medication-related, patient-related, acceptance, questionnaire,..etc • Mention the type of statistical tests applied | Thank you for the comments; we have revised the abstract accordingly. We have clearly stated the knowledge gap, specifying the limited evidence on oral solid medication acceptance among elderly patients in LMICs. The type of data analyzed is now clarified, including medication-related characteristics, patient demographics, clinical and behavioral factors, and acceptance measured via structured questionnaires. We also added the statistical tests applied, specifying bivariable and multivariable logistic regression with cluster-robust standard errors to account for multiple medications per patient. |
|  | **Methods:Details** about sample size calculation and sampling technique could be moved to the suppl. Material. | Thank you for the comment; we have moved the detailed description of sample size calculation and the sampling technique to the supplementary material, while keeping a concise summary in the Methods section for clarity. |
|  | **Results:** Age distribution with min and max to present in the demographic table. No supporting information is available. Provide the applied questionnaire as suppl. Material. | Thank you for the comments; we have updated the demographic table to include age distribution with minimum and maximum values. Additionally, the full questionnaire used for data collection has been provided as supplementary material. |
|  | **Discussion**  The results are largely expected, could the authors discuss what their study add to the existing literature or which particular aspects were deduced regarding the setting in a low-middle income/resource country? For the problematic dosage forms, were there other alternatives on the market that were easier to swallow e.g. smaller or dispersible, that the physician may prescribe? Were there any results describing patient experience with powders? Soft gelatine capsules? To be discussed. How was the OSDF distribution?  The results of the study regarding the size and coating should also be discussed in light of the feasibility and limitations of the pharmaceutical manufacturing process. The authors should acknowledge in their discussion not only patient preferences but also constrains by manufacturing feasibility such as dose load, coating materials, compression strength ..etc. | Thank you for the comments; we have revised the Discussion accordingly.   - We now clearly highlight the study’s contribution to the literature, emphasizing context-specific insights from a low- and middle-income country (LMIC) setting, where formulation options and healthcare infrastructure are limited. - We clarified that the study only included tablets and capsules (both hard and soft gelatin), and no powders without reconstitution were used, so patient experience with powders could not be assessed. The distribution of oral solid dosage forms (OSDFs) is now described. - We discussed alternatives such as smaller tablets, dispersible formulations, and soft gelatin capsules, noting their limited availability in this setting. - The discussion of size and coating has been expanded to include manufacturing feasibility considerations: dose load, coating machinery, raw materials, compression strength, and stability constraints, and we note the need to balance patient preferences with technical and cost limitations in LMICs. - Strategies such as prioritizing commonly used medications for patient-centered design or exploring multi-particulate and film-coated high-dose systems have also been added to reconcile patient needs with manufacturing feasibility. |
|  | PLOS authors have the option to publish the peer review history of their article ([what does this mean?](https://track.editorialmanager.com/CL0/https:%2F%2Fjournals.plos.org%2Fplosone%2Fs%2Feditorial-and-peer-review-process%23loc-peer-review-history/1/010f0199dd1524a7-3971c8ef-7ee2-4bae-881b-9bab77de2ec9-000000/y4-UbsntYdviKyPcMgCdKbFQA9uLYRtGy4ncvmFQRNg=232)). If published, this will include your full peer review and any attached files. | **Still not** |
