# Supplementary material for: The need for patient-centric medicine design: investigating key physical characteristics of oral solid medications to improve acceptance of older patients in Addis Ababa, Ethiopia
Source: PLoS One. 2026 Mar 6;21(3):e0331267. doi: 10.1371/journal.pone.0331267 (PMC13046391; doi:10.1371/journal.pone.0331267)
Supplement: Attachment — Submitted filename: Response_to_Reviewers_auresp_2.docx [file pone.0331267.s006.docx]

**Response to Reviewers**

**Dear Reviewers and Editors,**

Thank you for your constructive feedback and valuable suggestions. We have addressed all the comments and questions rose in the revised manuscript and provided detailed responses in the separate rebuttal document.

| **S/N** | **Question/comment** | | | **Response** |
| --- | --- | --- | --- | --- |
|  | **Introduction**   - Please be more specific in the introduction which aspects were assessed in the questionnaire and summarized under the term acceptability. - Please also explain more clearly why you expect that the results in Ethiopia differ from those reported for Europe. | | | Thank you for your constructive comments. We have revised the Introduction accordingly and addressed all the issues raised. In particular, we clarified the specific dimensions of medication acceptability assessed in this study.  Regarding the second comment, the rationale for expecting differences between findings from Ethiopia and those reported in European studies was strengthened based on the recommendations of Shariff et al. (2020). This study highlighted that existing European research does not adequately represent Black populations and may not fully capture the influence of sociocultural, healthcare system, and formulation-related factors relevant to low-resource settings. Our study was therefore designed in line with these recommendations to generate context-specific evidence and address this important gap in the literature. |
|  | **Methods and material** | | General | Thank you for the comment; have reviewed the method and material part in detail and we found most should be correct and did accordingly. |
|  |  |  | What exactly do you mean by “Eligible participants …, based on patient discharge order.”? | Thank you for highlighting this point. By “eligible participants were selected … based on patient discharge order,” we meant that systematic random sampling was applied using the outpatient service flow. After patients completed their clinical consultation and received their medications from the hospital pharmacy, every K^th^ eligible elderly patient was approached for participation according to the order in which patients exited the outpatient department. This wording has been revised in the Methods section to improve clarity. |
|  |  |  | It is also unclear what you mean by “...those who held some of his/her medicine, and those who were volunteers,...”? | Thank you for pointing out this ambiguity. By “those who held some of his/her medicine,” we meant elderly patients who were able to physically present at least one of their currently prescribed oral solid medications at the time of the interview, allowing direct assessment of medication characteristics. “Those who were volunteers” refers to patients who voluntarily agreed to participate after being informed about the study. The wording has been revised in the Methods section to improve clarity. |
|  |  |  | What do you mean by “A maximum of 5 oral solid medications were taken from the elderly patients, which can cover more than 90 percent of their medications.”? | Thank you for requesting clarification. By this statement, we meant that for each participant, up to five currently used oral solid medications were selected for assessment. This limit was applied to reduce participant burden and interview duration, while still capturing the majority of medications used by elderly patients. Previous evidence indicates that evaluating up to five medications per patient accounts for more than 90% of chronically used medicines among elderly populations. The Methods section has been revised to clarify this point. |
|  |  |  | Have you checked that those variables, which you call independent, are not correlated to each other? For instance, education, income and residence or certain symptoms and diseases. | Yes, potential correlations among independent variables were assessed during the analysis. Conceptually related variables (e.g., education, income, occupation and others) were first reviewed for overlap, and statistical multicollinearity was evaluated using variance inflation factors (VIFs) in the regression models. All variables included in the final multivariable analysis had VIF values within acceptable limits, indicating no problematic multicollinearity. This clarification has been added to the Methods section. |
|  |  |  | The terminology used in the text here, e.g. palatability is not used in the questionnaire itself. Did you use taste and other aspects to come up with a palatability score? If yes, how was this done? | Thank you for this observation. The term palatability was used in the manuscript as a conceptual descriptor rather than as a direct questionnaire item. In the questionnaire, palatability-related aspects were assessed using specific items such as “…tastes good” and “….has no bad aftertaste.” These items were analyzed individually and, where appropriate, combined as part of the overall medication acceptability score derived from the MAQ-2019, rather than as a separate standalone palatability score. |
|  |  |  |  |  |
|  |  |  |  |  |
|  | **Results and discussion** | Figure 1: How representative is your study population compared to the population in Ethiopia in the same age range? | | Thank you for this comment. The study population was drawn from public tertiary and general hospitals in Addis Ababa, which serve as national referral centers and attract elderly patients from both urban and rural areas across Ethiopia due to the availability of better diagnostic and treatment services. Importantly, although the study was conducted in Addis Ababa, only 34.1% of the study participants were residents of Addis Ababa, while the majority originated from other regions of the country. This reflects the national referral role of these hospitals and enhances the geographic diversity of the study population. |
|  |  |  | | As a result, the age and sex distribution of participants is broadly comparable to the national elderly population, and the sample captures a wide range of chronic conditions and medication-use experiences from different parts of Ethiopia. While we acknowledge that the study is facility-based and may not represent elderly individuals who do not access hospital services, we believe it provides a reasonable representation of healthcare-utilizing elderly populations at the national level. |
|  |  | The terms in the questionnaire are often not clearly defined, such as swallowability difficulties, visual impairment, …Furthermore, looking at the questionnaire and the results I wonder how you managed in multimorbid patients using polypharmacy to distinguish if the amount of medications are attributed purely to the disease. Please explain how you did this, e.g. “Among the disease conditions affecting elderly patients, hypertension emerged as the most common (43.1%) with 50.2% of oral solid medication usage, followed by other cardiac-related disorders and diabetes mellitus.” | | First, key terms used in the analysis were operationalized through specific questionnaire items rather than as single, abstract concepts. For example, swallowability difficulties were assessed using multiple patient-reported items, including whether the medication: (i) feels stuck in the throat, (ii) requires excessive effort or repeated swallowing, (iii) causes choking or coughing during swallowing, and (iv) requires additional water or medication modification (e.g., crushing or splitting) to swallow. Visual impairment was similarly assessed using self-reported difficulty in reading medication labels, distinguishing tablets, or identifying score lines without assistance. Second, regarding multimorbidity and polypharmacy, we did not attempt to attribute individual medications to specific disease indications. Instead, patients’ chronic medical conditions (e.g., hypertension, diabetes, cardiac disorders) were identified from medical chart reviews, while information on medications was obtained from patient interviews and direct inspection of medicines. The reported result that 43.1% of patients were hypertensive and that these patients accounted for 50.2% of all oral solid medications used indicates that hypertensive patients many of whom also had additional chronic conditions were using a disproportionately higher number of medications overall. Importantly, this does not imply that 50.2% of medications were prescribed specifically for hypertension.  Each patient contributed all of their currently used oral solid medications to the analysis, regardless of indication. This approach reflects real-world medication use in elderly patients with multimorbidity and polypharmacy. We have revised the Methods and Results sections to clarify this point and prevent misinterpretation. |
|  |  | How was the taste assessed? How reliable is the result communicated based on that? Table 5 mentioned “bitter color” as taste and “partial score line”. What is meant here? | | Thank you for raising this point. Taste was assessed using patient-reported perceptions rather than objective sensory testing. Specifically, participants were asked to rate statements such as “the medicine tastes good” and “the medicine has no bad aftertaste” using a Likert-scale format. These questions captured the patient’s subjective experience at the time of medication intake. While taste perception is inherently subjective, patient-reported assessment is considered appropriate and reliable for evaluating medication acceptability, particularly in elderly populations, as it reflects real-world use. The internal consistency of the acceptability-related items, including taste-related questions, was acceptable (Cronbach’s α = 0.77), supporting the reliability of the reported results.  Regarding Table 5, the term “bitter color” was a wording error and did not refer to taste. It has been corrected to “bitter taste” in the revised manuscript. The term “partial score line” refers to tablets with a score line that does not fully divide the tablet into equal halves or is not intended to ensure dose accuracy but primarily facilitates breaking for ease of swallowing. Both terms have now been clearly defined in the Methods and Operational Definitions sections to avoid confusion. |
|  |  | How was the judgement on the labelling performed? Was language taken into account? | | Labeling assessment took language into account; however, we clarify that none of the medications were labeled in Amharic. Judgement of labeling was therefore based on the patient’s ability to read and understand English-language labels, including instructions, font size, and clarity of information. Patients who reported being able to read and understand English labels had better labeling acceptance, whereas those with limited or no English proficiency reported greater difficulty. |
|  |  | From a methodological point of view I have some issues understanding based on the questionnaire, how the column acceptance of medication in table 6 was derived. It seems like “acceptance” yes or no is a value related to the patient and not the medicine he/she takes.  This makes it difficult to understand the conclusions made such as the score lines are better accepted than tablets without. Might be an effect of reimbursement (prescribing a higher dose strengths) and asking the patient to split medication. In this context you should discuss dosing errors etc. | | Medication acceptance was assessed at the medication level, not solely at the patient level. Although the questionnaire was administered to patients, acceptance responses were collected separately for each oral solid medication currently used by the patient, up to a maximum of five medications per participant. For each medication, the MAQ-2019 items were answered with reference to that specific medicine (e.g., ease of swallowing, handling, taste, appearance, labeling, and willingness to take without modification). A composite acceptability score was calculated for each medication, and this score was dichotomized into “accepted” versus “not accepted” using the predefined threshold. Therefore, the acceptance outcome presented in Table 6 represents medication-level acceptability, while accounting for clustering of multiple medications within individual patients. This has been clarified in the Methods section.  We agree that acceptance of score-lined tablets may be influenced by prescribing practices, including higher dose strengths and the need for tablet splitting. However, in our study, the presence of a score line was evaluated from the patient perspective, specifically whether it facilitated easier handling or swallowing and reduced the need for uncontrolled breaking. We did not evaluate reimbursement-related prescribing decisions, as these are not applicable in the Ethiopian public healthcare context where medicines are largely subsidized or provided at low cost. We acknowledge that tablet splitting may introduce dosing inaccuracies, and this potential risk has now been explicitly discussed in the Discussion section as an important consideration when interpreting the higher acceptance of score-lined tablets. |
|  |  | In the discussion you mention limitations of medications in Ethiopia, e,g. Not available in a different format (dispersible, with score line, …). Are those medications available in a different format in other countries / elsewhere? | | Regarding the availability of alternative formulations, such as dispersible tablets or age-adapted dosage forms, these formulations are commonly available in high-income countries and are often recommended by regulatory agencies (e.g., EMA) as part of patient-centered medicine design for elderly populations. In contrast, such alternatives are limited or largely unavailable in Ethiopia, where the market is dominated by standard generic solid dosage forms. We have revised the Discussion section to explicitly contrast the Ethiopian context with international availability and regulatory practices. |
|  |  | Looking at the questionnaire itself it would have offered other important insights into dosing frequency, easy to take (with/without food),...Unfortunately those aspects are not mentioned in the article. | | We acknowledge that the questionnaire included additional domains such as dosing frequency and ease of administration in relation to food. These variables were collected primarily as contextual and descriptive information and were not the primary focus of the current analysis, which was specifically designed to investigate physical formulation-related characteristics of oral solid medications. To maintain focus and avoid overextension of the manuscript, these aspects were not included in the final analysis. This has now been acknowledged as a limitation in the Discussion section. |
